# The interrelationship between the face and vocal tract configuration during audiovisual speech

**DOI:** 10.1073/pnas.2006192117

**Published:** 2020-12-08

**Authors:** Chris Scholes, Jeremy I. Skipper, Alan Johnston

**Affiliations:** ^a^Visual Neuroscience Group, School of Psychology, University of Nottingham, NG7 2RD Nottingham, United Kingdom;; ^b^Experimental Psychology, University College London, WC1H 0AP London, United Kingdom

**Keywords:** audiovisual, speech, PCA

## Abstract

It is well established that speech perception is improved when we are able to see the speaker talking along with hearing their voice, especially when the speech is noisy. While we have a good understanding of where speech integration occurs in the brain, it is unclear how visual and auditory cues are combined to improve speech perception. One suggestion is that integration can occur as both visual and auditory cues arise from a common generator: the vocal tract. Here, we investigate whether facial and vocal tract movements are linked during speech production by comparing videos of the face and fast magnetic resonance (MR) image sequences of the vocal tract. The joint variation in the face and vocal tract was extracted using an application of principal components analysis (PCA), and we demonstrate that MR image sequences can be reconstructed with high fidelity using only the facial video and PCA. Reconstruction fidelity was significantly higher when images from the two sequences corresponded in time, and including implicit temporal information by combining contiguous frames also led to a significant increase in fidelity. A “Bubbles” technique was used to identify which areas of the face were important for recovering information about the vocal tract, and vice versa*,* on a frame-by-frame basis. Our data reveal that there is sufficient information in the face to recover vocal tract shape during speech. In addition, the facial and vocal tract regions that are important for reconstruction are those that are used to generate the acoustic speech signal.

## Significance

Speech perception is improved when we are able to see the person who is speaking, but how visual speech cues are used to improve speech perception is currently unclear. Brain imaging has revealed that regions responsible for motor control are active during the perception of speech, opening up the possibility that visual cues are mapped onto an internal representation of the vocal tract. Here, we show that there is sufficient information in the configuration of the face to recover the vocal tract configuration and that the key areas responsible for driving the correspondence vary in accordance with the articulation required to form the acoustic signal at the appropriate point in a sentence.

While speech is predominantly an acoustic signal, visual cues can provide valuable information about the timing and content of the signal. These visual cues become increasingly important when the acoustic signal is degraded, for example, in individuals with hearing problems (1, 2), or when the signal needs to be extracted from noise (3) or mixed competing speech sources, as may occur at a cocktail party (4). Audiovisual speech integration is predominantly discussed in the context of comparing high-level acoustic and visual representations. For example, integration has been framed as a temporally focused lexical competition, in which visual information is used to constrain the corpus of words indicated by the acoustic signal (5). While the neural loci of audiovisual speech integration have been studied extensively (6–11), the exact nature of the visual representation utilized in audiovisual speech integration remains unclear (12). Here, we address the questions of whether and how visual speech signals might provide information that is compatible with acoustic speech signals.

One possibility is that visual speech cues are mapped onto an internal representation of the speech articulators before being combined with compatible representations derived from auditory information. There is a large amount of evidence for a ubiquitous role for sensorimotor systems in auditory and audiovisual speech perception (13). These results are more consistent with theories of speech perception in which contextual information across modalities is used to parse incoming speech [e.g., analysis by synthesis (14)], than either unisensory (15) or motor (16, 17) theories. In analysis by synthesis, a preliminary analysis is done making use of contextual information “derived from analysis of adjacent portions of the signal” which can presumably include visual information (18, p. 99). These are then used to derive the underlying motor commands used to produce that information which are, in turn, used in a predictive manner to constrain interpretation of acoustic information arriving in auditory cortex. Analysis by synthesis in particular, in that it contains a model of articulators, provides a clear focus of integration of auditory and visual information. This type of integration would require visual speech information to covary with information about both observable and unobservable articulators during speech production.

Here, principal components analysis (PCA) was applied to combinations of frontal image sequences of faces and sagittal fast magnetic resonance (MR) image scans of the vocal tract to assess the extent to which facial speech cues covary with articulator dynamics. PCA has been applied to still images of multiple individuals for facial recognition (19–21) and to sequences of images to highlight regions that are important for dynamic face perception (22). It has also been used to quantify the degrees of correlation between user-defined points on the face and vocal tract during speech production (23). Our approach extends this previous work by applying PCA to whole-image sequences of the face and vocal tract during speech sentence production, rather than specifying regions of interest a priori. A clear benefit to the use of vocal tract MR imaging over electromagnetic articulography (EMA), which has been used in earlier work, is that it avoids the need for sensors on the tongue, velum, and lips, which may influence articulation. In addition, both the face and vocal tract are densely sampled, and recent work demonstrates that high-resolution cues are important during audiovisual speech perception (24). MR imaging does, however, lack the temporal resolution of EMA (25). PCA operates as a kind of autoassociative memory (26), allowing occluded regions of its inputs to be reconstructed (e.g., 21, 22). We leveraged this feature of PCA to recover vocal tract sequences from facial video sequences and show that this approach results in reconstructions with high fidelity when compared to the original (ground truth) vocal tract sequence. This demonstrates that an unsupervised learning strategy can be used to recover the whole vocal tract from the face alone and that this could, in principle, be used as an internal model during the process of speech perception.

## Results

### Reconstructing MR Images of Speech Production.

**Fig. 1.**
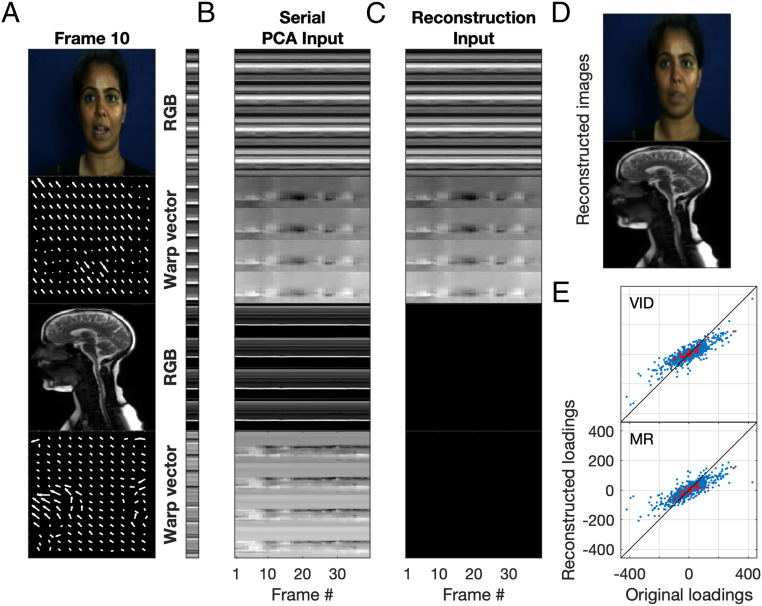
Overview of image sequence reconstruction after application of PCA to hybrid video–MR images. (*A*) Example frame for actor 8 and sentence 1. Frame images are shown for both facial video and MR sequences, with a depiction of the warp field necessary to transform this image to the reference image shown in the panel underneath. The bar to the right shows the 1D serial vector for a slice through this frame, taken column-wise from the RGB pixel values in the image and the *x* and *y* warp vectors. (*B*) Serial vectors are concatenated across frames to create a 2D array which acts as the input to the PCA (slices through each frame are depicted here, for display purposes, but the full frames were used in the PCA input). (*C*) One modality was reconstructed using the input for the other modality and the PCA. The illustration shows the values for the MR modality have been set to zero. This array was then projected into the PCA space, and the MR sequence was reconstructed. In the same way, but not depicted here, the video sequence was reconstructed from the MR sequence and the PCA. (*D*) Reconstructed images for the example frame shown in *A*. (*E*) Reconstructed loadings as a function of the original PCA loadings for all frames (blue dots) and for the example frame (red dots), with the reconstructed modality indicated in each panel (VID = facial video).

PCA was performed on hybrid arrays of MR scans and frontal facial videos of individuals repeating the same sentence, as detailed in *Materials and Methods*. Fig. 1*A* shows a frame from the facial video and MR sequence for an example actor (actor 8) and sentence (sentence 1: “Miss Black thought about the lap”), along with the vector field describing how to warp this frame to the reference frame (see *Materials and Methods*). Full-sequence videos can be found in Movies S1–S9. The PCA captured regions of the face and vocal tract that changed during the sentence, for example, the mouth and tongue, effectively ignoring features, such as the brain and spinal cord, which remained stationary. As PCA operates as a kind of autoassociative memory, MR sequences could be reconstructed by projecting just the video input data (Fig. 1*C*) into the PCA space, and video sequences could be reconstructed by projecting just the MR data into the PCA space. Differences between the original (Fig. 1*A*) and reconstructed MR sequences (Fig. 1*D*) were subtle and resulted from an underestimation of facial or vocal tract movement (for the full sequence, see Movie S10). This was reflected in the reconstructed loadings, which can be interpreted as the degree of similarity of the reduced input vector to each of the principal components (PCs) (Fig. 1*E*). Since the magnitude of the vector is reduced, and the loading reflects the projection of the reduced vector onto the PCs, the reconstructed loadings will always be smaller in magnitude than the original loadings. Irrespective of these small differences in magnitude, the loading correlation was high (MR reconstruction: Pearson’s *R* = 0.85, *P* *<* 0.01; video reconstruction: *R* = 0.87, *P <* 0.01), indicating that the frame sequences for both modalities could be reconstructed with high fidelity by projecting the input data for the other modality into the PCA space.

**Fig. 2.**
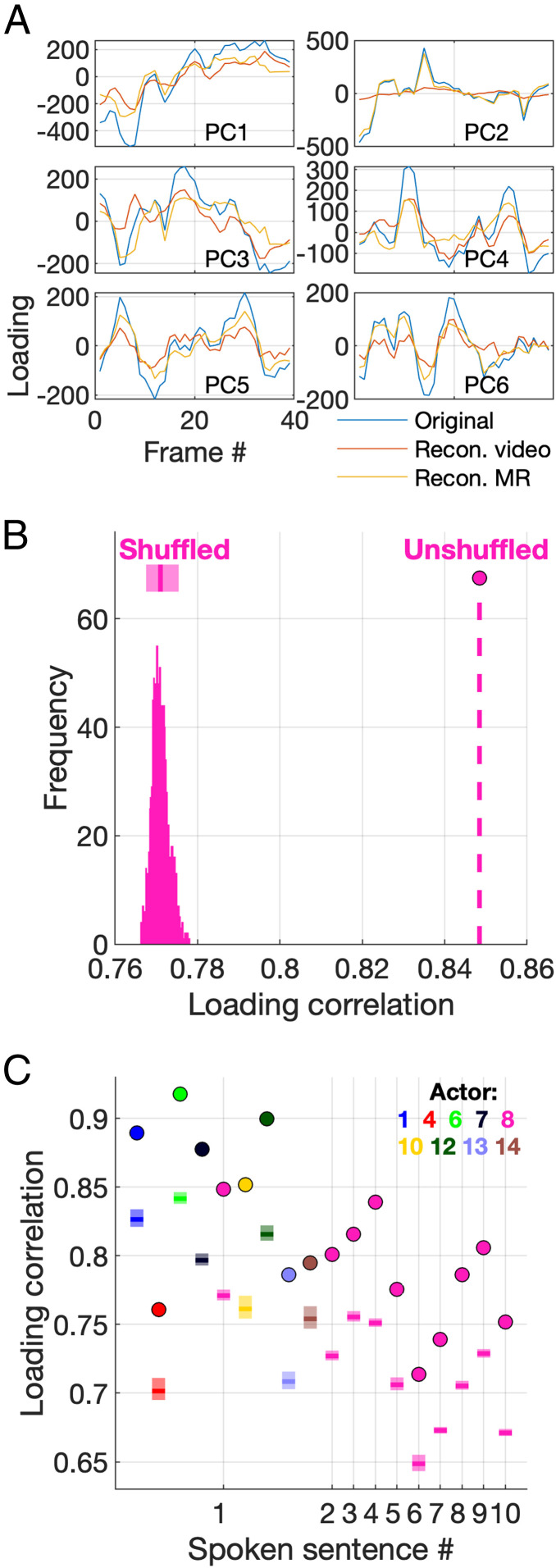
The ability to reconstruct vocal tract images from the PCA is dependent on the correspondence between the configurations in the video and MR sequences. (*A*) Loadings for the first six PCs across all frames for the example actor and sentence for the original sequence (blue) and both the reconstructed MR (orange) and video (gold) sequence. (*B*) Loading correlation for the example actor and sentence for the original MR reconstruction (dashed line) and the distribution of loading correlations for 1,000 permutations of randomized MR frame order. The mean (solid bar) and 95% confidence intervals (lighter bars) are indicated above the distribution. (*C*) Original loading correlation (circles) and shuffled loading correlation mean (solid bar) and 95% confidence intervals (lighter bars) across all sentences for one actor and one sentence (sentence 1) for nine actors.

Delving deeper into the PCA representation, Fig. 2*A* shows how the loadings for the first six PCs vary on each frame for each reconstructed modality. For the majority of the PCs, the reconstructed loadings vary in a similar way to the original loadings, albeit at a lower magnitude. This suggests that these PCs account for feature variation that is shared across the two modalities, and this can be visualized in videos reconstructed for each individual PC (Movie S11). In this example, PC 2 exclusively accounted for variation in the MR sequence; however, as indicated by the high loading correlation, most PCs accounted for shared variation across the two modalities. An interesting feature of this approach is that the sum of the reconstructed loadings for each frame and PC exactly equals the original loading, such that the reconstructed loadings differ only by a scaling factor from the original loadings. While this scaling factor varies somewhat across frames and PCs, loadings can be augmented by applying a mean scaling factor, computed across all PCs and frames. While this seems a reasonable step, given that removing one of the modalities from the original PCA input has, in effect, halved the amount of data that is used to reconstruct that modality, we did not scale the data in this paper.

We have demonstrated that MR sequences of sentence production can be reconstructed with high fidelity from a PCA applied to videos of individuals uttering the same sentence, and vice versa. We would like to say that the reconstruction fidelity reflects the shared variance in the data arising from the fact that the variation of appearances in the video and MR pairings have the same cause—the articulation required to generate the speech signal. However, it is possible that the relationship arises from the mere, arbitrary, association of pairs of video and MR frames. To explicitly test this, we shuffled the order of the MR images while maintaining the correct order of the video images and then performed PCA on these shuffled hybrids (we targeted the reconstruction of MR sequences from video sequences, which focuses on the question of the recovery of the vocal tract configuration from the facial configuration). Fig. 2*B* shows, for our example sentence and actor, the distribution of loading correlations from 1,000 permutations of MR frame order and the loading correlation for the original (and correct) frame order. Even after shuffling the temporal order of the MR images with respect to the video, those MR images could be reconstructed with a high fidelity given the video images (in the correct order) and the PCA. Importantly, however, the reconstruction fidelity for the correctly ordered frames was always higher than the fidelity for shuffled permutations. The pattern observed for the example sentence was conserved across all sentences and across nine actors (Fig. 2*C*). The correlation for the correctly ordered MR sequence was always significantly higher than for the shuffled sequences (for a permutation test with 1,000 iterations, *P <* 0.001). In addition, the sum of squared errors (SSE) between the original and reconstructed loadings for the correctly ordered sequence was always significantly lower than for the shuffled sequences (*SI Appendix*, Fig. S1), providing further evidence that reconstructions were more faithful to the original sequence when the frames were in the correct temporal order (for a permutation test with 1,000 iterations, *P <* 0.001). Taken together, this indicates that the ability to reconstruct MR images from the PCA is dependent on the correspondence between the configurations in the video and MR sequences, signifying that PCA is able to encode something of the common cause that leads to the joint appearance, over and above mere association.

### Explicitly Including Temporal Information in the Observations on Which the PCA Is Performed.

**Fig. 3.**
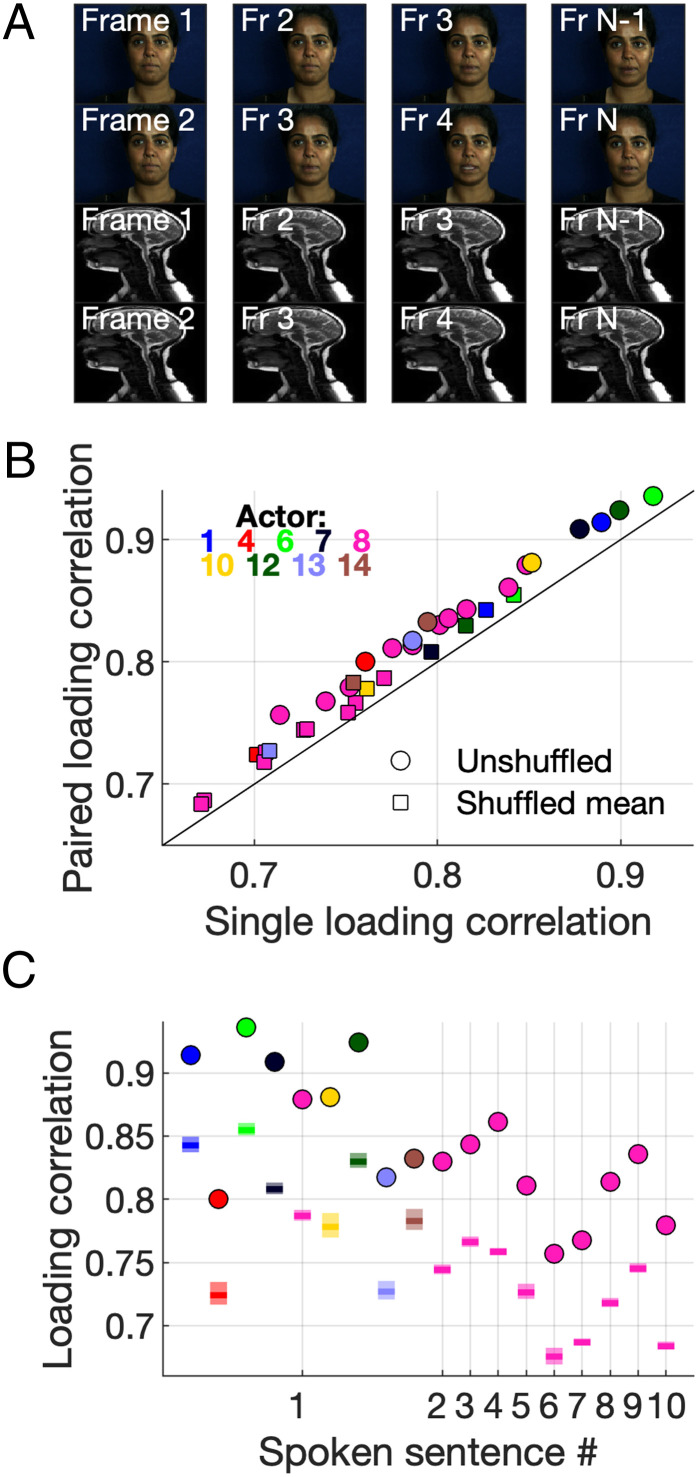
Implicitly including temporal information in the PCA input leads to increased reconstruction fidelity. (*A*) Paired-frame PCA input. (*B*) Paired-frame loading correlation as a function of single-frame loading correlation for unshuffled (circles) and shuffled (squares) sequences. (*C*) Original paired-frame loading correlation (circles) and shuffled paired-frame loading correlation mean (solid bar) and 95% confidence intervals (lighter bars) across all sentences for one actor and one sentence for nine actors. Actor color code used in this figure is identical to that used in Fig. 2*C*.

In our implementation, each (matched) frame within the hybrid MR–video data array can be considered as a sample, and the pixel and warp values for each frame are observations. PCA acts to define components that maximize the variance explained across these observations, and because they solely describe spatial variations, temporal variations across frames are ignored. The order of the vectors could be shuffled without affecting the outcome of the reconstruction. In practice, this means that our PCA has no information about the direction of motion. For example, an open mouth on any given frame could be the result of the mouth opening or closing; however, the PCA would be ignorant of this information in our original implementation. To investigate the effect of implicitly including temporal information in our hybrid representations, we included both the current frame and the next frame as observations for each sample (see *Materials and Methods* and Fig. 3*A*).

Reconstruction fidelity, quantified using either the loading correlation (circles in Fig. 3*B*, one-tailed *t* test, degrees of freedom [df] = 17, *P <* 0.01) or SSE (one-tailed *t* test, df = 17, *P <* 0.01), increased significantly when temporal information was included, for one sentence uttered by nine actors and across all sentences uttered by our example actor. We employed the same shuffling technique as previously, but maintained frame-by-frame pairings. Again, the original correctly ordered sequence could be reconstructed with a higher fidelity than all of the 1,000 randomly shuffled sequences (Fig. 3*C*, for a permutation test with 1,000 iterations, *P <* 0.001). Generally, the increase in mean loading correlation for the randomly shuffled paired sequences over the shuffled single-frame sequences (squares in Fig. 3*B*) was smaller than the increase for the unshuffled sequences noted above. One interpretation is that implicit representation of temporal order across the observations makes the paired sequences more robust to shuffling across the sample dimension. We tested this by randomly shuffling the frame order before pairing the frames, thus removing any frame-by-frame dependencies, which led to an increase in shuffled loading correlations that was more in line with that observed in the unshuffled case (*SI Appendix*, Fig. S2).

In summary, implicitly including temporal information in the PCA model leads to increased reconstruction fidelity and a greater robustness in the face of frame-order shuffling.

### Which Regions of the Face and Vocal Tract Are Important for Reconstruction?

**Fig. 4.**
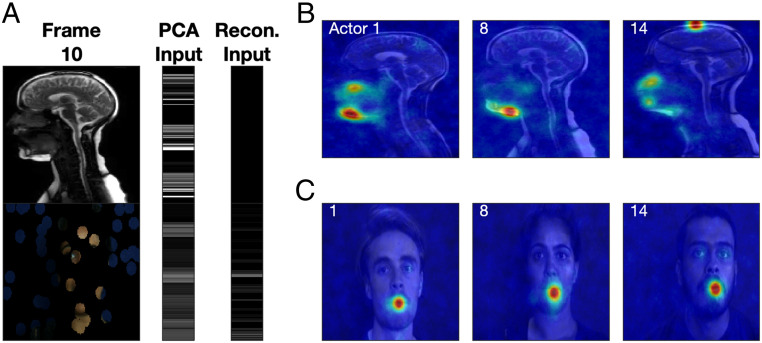
A Bubbles analysis reveals the regions of the vocal tract that are important for reconstruction of the face and vice versa, across the whole sentence. (*A*) Example frame for actor 8 and sentence 1, with a random bubble mask applied to the single video frame (mask was also applied to the warp vector fields which, for clarity, are not depicted here). The bars to the right show the 1D serial vector for this frame, taken column-wise from the RGB pixel values in the image for the original PCA input (*Left* bar) and once the bubble mask has been applied to the reconstruction input (*Right* bar). (*B*) *ProportionPlanes* overlaid onto the first frame from the MR sequence (for display purposes only) for three randomly selected actors. (*C*) *ProportionPlanes* overlaid onto the first frame from the video sequence (for display purposes only) for the three actors. In both *B* and *C*, the hotter the color, the more that region contributed to the top 10% of reconstructions, based on the loading SSE.

Bubbles analysis was used to interrogate the underlying PCA representation by testing which regions of a sequence of images for one modality are important for the reconstruction of the image sequence for the other modality. A mask of randomly positioned discs was applied to the images and vector fields for one modality, this vector was projected into the original PCA space, and the frame sequence for the other modality was reconstructed as before (Fig. 4*A*). This process was repeated 10,000 times with a new set of random bubble positions selected each time. Reconstruction fidelity (quantified using loading SSE) was then used to select out the top 10% of reconstructions, and the masks for these reconstructions were summed and divided by the sum of all masks to give a *ProportionPlane* (see *Materials and Methods*).

The *ProportionPlanes* for the example sentence uttered by three randomly selected actors are displayed in Fig. 4*B* (the “heat” indicates the regions of the MR images which are important for facial video reconstruction) and Fig. 4*C* (the “heat” indicates the regions of the video images which are important for MR vocal tract reconstruction). The Bubbles analysis reveals that, across actors, the gross region around the upper and lower jaw in the vocal tract scans is important for facial video reconstruction (Fig. 4*B*). Similarly, the mouth region dominates when we consider areas in the facial video that are important for vocal tract reconstruction (Fig. 4*C*).

**Fig. 5.**
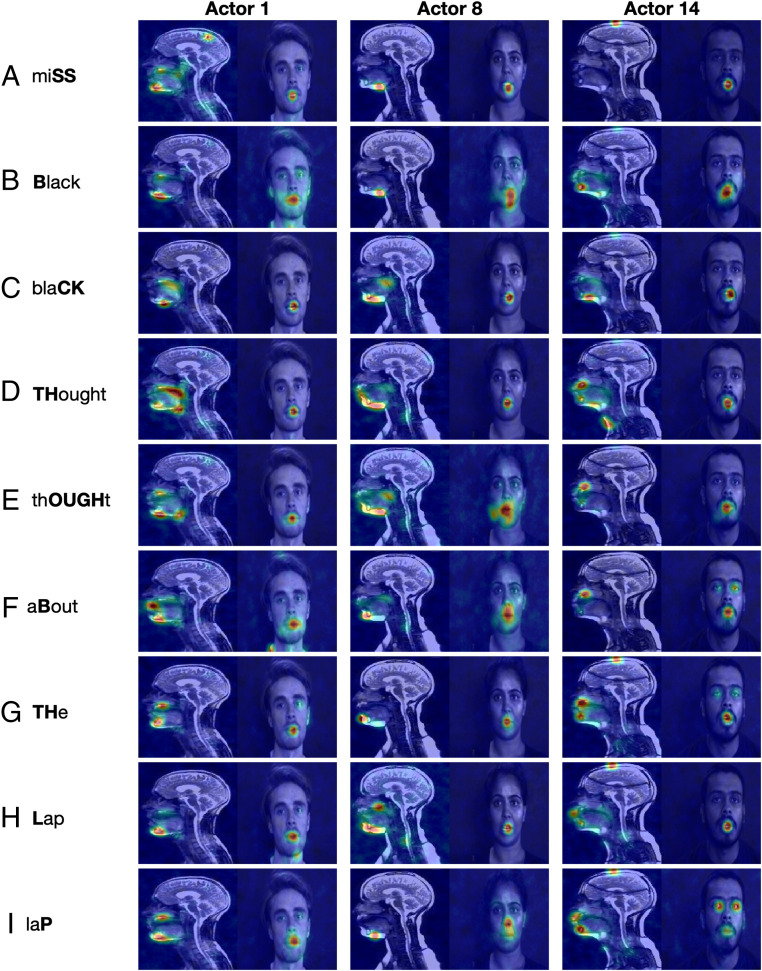
Frame-by-frame Bubbles analysis for selected phrases in the example sentence (indicated in bold beside each row) for three randomly selected actors (indicated above each column). *ProportionPlanes* overlaid onto each frame: the hotter the color, the more that region contributed to the top 10% of reconstructions for that frame, based on the loading SSE.

Fig. 4 illustrates the regions of the face and vocal tract that are most important for reconstruction with respect to the sequence taken as a whole; however, this masks any time-dependent relationship that is related to the speech. A frame-by-frame analysis reveals that the areas that are important for reconstruction follow a more intricate and dynamic pattern as the sentence unfolds (Fig. 5; full-sequence videos can be found in Movies S12–S20). Regions of the vocal tract that are important for reconstructing the face vary depending on the sound that is being created. For example, the upper and lower lips are important for reconstructing the face when there is a plosive, such as the “B” in “Black” (Fig. 5*B*) and “aBout” (Fig. 5*F*), or the “P” in “laP” (Fig. 5*I*). Similarly, the back of the oral cavity is important for the “CK” in “blaCK” (Fig. 5*C*), when the tongue moves up toward the velum. The extent of the facial oral region that is important for vocal tract reconstruction varies during the video sequence. Additionally, along with the mouth, extraoral features such as the eyes (e.g., actors 1 and 14 in Fig. 5 *F*, *G*, and *I*) and cheeks (as they are drawn in during the “OUGH” in “thOUGHt” for actor 8 in Fig. 5*E*) are highlighted.

## Discussion

Using an application of PCA, we have shown that the configuration of the face can be recovered from that of the vocal tract during speech production, and vice versa, adding to previous work which applied PCA to motion recordings from selectively placed markers on the face and vocal tract during speech (23). Building on this knowledge, we showed that the fidelity of the recovered frame sequences was dependent on the temporal correspondence between the two modalities, suggesting that the PCA is capturing a common cause rather than merely the association between the face and vocal tract images. By including paired frames in each sample we also showed that knowledge of the direction of articulator motion is perhaps beneficial in disambiguating, for example, whether the mouth is opening or closing during a particular phrase. From Yehia et al.’s work (23), we knew that there was a general correspondence between facial and vocal tract features over the course of a sentence—the “coupled motion” of the jaw, tongue, and lips. An important step here was to show that facial and vocal tract covariation is connected specifically during particular phases of the sentence, rather than simply being generally linked throughout the course of the sentence. An example of this is the importance of the velum in the vocal tract representation during the “CK” of “blaCK,” when the tongue is withdrawn and raised toward the back of the mouth. Specifically, recovered facial images were closer to the originals when the velum region of the vocal tract image was projected into the PCA space as compared to other regions of the vocal tract. And importantly, this was true for the images relating specifically to the part of the sentence (the “CK” in “blaCK”) where the tongue moved back toward the velum.

Recall that the acoustic signal was not included in our PCA model, so the associations that were captured by the PCA were purely based on the images of the face and the vocal tract during speech production. Nevertheless, the face and vocal tract configurations were intrinsically connected, being those required to produce a specific speech sound. Our work aligns with theories on audiovisual integration based on joint causation. Three modalities (the face, vocal tract, and sound) are all linked to a single cause, and because of this they share correlated variation (e.g., 27). Note that the relationship uncovered here is based on the configuration of both the face and the vocal tract, and therefore the principal link relates to how the face and the vocal tract are structured. This implies that the face provides information implicitly or explicitly about how the vocal tract is structured and how this structure changes during speech.

Given that speech recognition is modulated (28) and improved by having access to visual speech cues (3, 29), a comprehensive theory of speech perception must include an explanation of how visual speech cues are integrated with the acoustic signal. The main theories of speech recognition allow for the mapping of facial articulators to an underlying motor representation but vary in the importance assigned to such a mapping. Both the motor theory of speech perception (16, 17) and analysis by synthesis (14) posit an internal representation of speech that is used for both perception and production. The motor theory is underscored by a process in which multisensory speech cues are mapped onto speech articulators. For analysis by synthesis, articulator movements are the “distinctive features” that are used to synthesize predictions of speech which are then compared to the analyzed acoustic signal. Other theories of speech perception are less reliant on an explicit connection between the vocal tract and face. Fowler’s direct realist theory of speech perception (30) posits that the information available across modalities is reduced to an underlying set of articulatory primitives that explicitly code the distal event (in this case speech) holistically. How the articulatory primitives are arrived at is still an open question, but neuroimaging work strongly favors a ubiquitous role for sensorimotor systems (13). The work here provides an important step by revealing that the full vocal tract configuration, including hidden articulations, can be recovered from parts of the face.

Our approach demonstrated which parts of the facial image are most informative about vocal tract dynamics. Importantly, although the oral region dominated, extraoral regions such as the cheeks and eyes were also highlighted. Although further investigation would be necessary to confirm whether the eye movements were meaningful with respect to the speech, it is likely that the cheeks were conveying useful information: they were highlighted as important as they were drawn in during production of the “OUGH” of “thOUGHt.” This indicates that visual information about speech is not limited to the mouth region, adding to previous work (31), and so any application for which speech recognition is the goal should consider extraoral regions as well as the mouth. Interestingly, eye-tracking studies have demonstrated that the eye and mouth regions are also those that are most looked at during face-to-face speech, with the proportion of time spent looking at each being dependent on task demands. Individuals spend more time looking at the mouth region when the task is difficult, for example, when the target speech is presented in noise (32) or is in an unfamiliar language (33), and speechreading performance has been shown to be positively correlated with the time spent looking at the mouth in deaf and normal-hearing children (34). Lansing and McConkie (35) showed that the eyes were attended more when analyzing emotional or prosodic content and the mouth more when speech segmentation was the goal. The observation that some parts of the face carry more information than others may inform theories about where people look when encoding facial speech.

In summary, we show that there is information in the configuration of the face about the configuration of the vocal tract. In addition, the informative regions of the face and vocal tract vary in both time and space in a way that is consistent with the generation of the speech signal. This is an important proof of principle that the full vocal tract configuration can be reinstated from parts of the face, complementing neuroimaging evidence that sensorimotor systems are involved in speech perception.

## Materials and Methods

### Actors.

Data were collected from 13 actors who were paid an inconvenience allowance for their participation. Of the 13 actors, only native English speakers were used here (11 actors). The study was conducted under ethics approved by local departmental boards at both the University of Nottingham and University College London. Nine of the eleven native English actors gave signed consent to use their recordings for analysis and publication, and it is those nine actors whose data are presented here.

### Stimulus Materials and Procedure.

The raw data consisted of color videos of actors repeating a set of sentences and, in a different session, monochrome MR scans of the same actors speech-shadowing these sentences. Facial video was captured simultaneously from five angles, ranging from side-on to frontal, using Grasshopper GRAS-03K2C (FireWire) cameras (PointGrey; 640 × 480 pixels [px], 30 frames per second [fps], red, green, and blue [RGB] 24-bit px format) positioned using a purpose-built camera rig. Audio was captured simultaneously, and camera and microphone signals were collated on a Dell desktop computer. Actors were seated in front of a blue screen and read sentences from an autocue controlled by the experimenter.

Facial videos were collected in a single session, in which 10 sentences from the speech intelligibility in noise database (Table 1; and ref. 36) were repeated 20 times. These sentences are from a corpus in which intelligibility, keyword predictability, phonetic content, and length have been balanced. The order of the sentences was randomized, but the same order was used for each actor. Subsequently, MR scans were collected across four runs within which the 10 sentences were repeated 20 times. Speech-shadowing was used during the MR scan to ensure the actors repeated the sentences in a way that was as similar as possible to the timing of the video recording. Specifically, the audio recording from the video was played back to the actor through headphones as they lay in the scanner, and the actor was required to reproduce what they heard as closely as possible.

**Table 1.** The 10 sentences from the speech intelligibility in noise database used in this paper

1) Miss Black thought about the lap.
2) The baby slept in his crib.
3) The watchdog gave a warning growl.
4) Miss Black would consider the bone.
5) The natives built a wooden hut.
6) Bob could have known about the spoon.
7) Unlock the door and turn the knob.
8) He wants to know about the risk.
9) He heard they called about the lanes.
10) Wipe your greasy hands on the rag.

### MR Imaging.

Vocal tract imaging was done on a 1.5T Siemens Avanto scanner at the Birkbeck–UCL Centre for Neuroimaging. T1-weighted anatomical images were collected with an acquisition time of 60 ms (sampling rate 16.67 Hz) over a single 10-mm midsagittal slice of the head and neck (2.125 × 2.125 mm, field of view = 256 × 256 mm, repetition time = 56 ms, echo time = 1.24 ms). Images were reconstructed using the Gadgetron framework and converted to audio video interleave (avi) for further processing.

### Hybrid Video Creation.

It is difficult to get an ideal view of the face in a scanner, and collecting multiple views from synchronized cameras would have been impossible. In addition, it would be a challenge to completely remove scanner noise from the speech signal. Thus, since facial video and MR scans were necessarily collected at different times, an initial alignment stage was required to create hybrid facial–MR frame sequences for each actor and sentence. First, the session-long facial video and MR scans were chopped into individual sentences using a combination of proprietary automated Matlab (Mathworks) scripts and ELAN annotation software (Version 5.5, Max Planck Institute for Psycholinguistics). We were able to leverage the output of the Multichannel Gradient Model (outlined below) during this process. Specifically, a reference image was selected to be as close to resting as possible. The output of the Multichannel Gradient Model (McGM) then described how far each pixel in each frame was from the resting face. For each actor, we selected a region of interest around the mouth/vocal tract and summed the McGM vectors for all pixels within this region. When viewed across the complete session recording, this vector peaked during each speech phrase, and we used the findpeaks function in Matlab to select out the 50 highest peaks. Custom Matlab code was used to cluster the frames from the speech phrase around each peak, and a video was created in which each phrase was highlighted with a false color. The videos were then inspected using the implay function in Matlab, and the start and end frames for each phrase were altered if the automated process had not been successful. This process yielded 20 facial videos and 20 MR scans for each sentence and actor. Second, each repeat was visually inspected, and the combination of facial video and MR that most closely matched in time was chosen. To aid in this process, videos containing all 20 video repeats and all 20 MR repeats were constructed using Matlab. These videos provided an initial insight into which combinations of facial and MR sequences would fit together best. Third, the start and end frame of the MR scan was selected such that the hybrid facial–MR videos were as closely aligned in time as possible, as assessed by visual inspection of the facial video and MR scan sequences presented side by side, again using custom Matlab code.

### Preprocessing of MR and Video Data.

Video images were reduced by 1/4 (from 640 × 480 to 160 × 120 px) using bicubic interpolation. MR images were not rescaled and had a resolution of 120 × 160 px. The PCA implementation required an equal number of frames in the video and MR sequences. Video was recorded at 25 fps, while MR images were acquired at around 16 fps; thus MR image sequences were always shorter than video sequences for the same utterance. To match the number of video frames for a particular combination of MR and video sequence, a scaling factor was calculated:

$$\text{scale factor}={\text{N}}_{\text{vid}}/{\text{N}}_{\text{MR}}$$

where N_vid_ was the number of video frames and N_MR_ was the number of MR images. The first frame and every rounded scaled frame were retained for further processing (e.g., if there were six MR frames and nine video frames, the scale factor would be 1.5, and the retained frame set would be round [1, 2.5, 4, 5.5, 7, 8.5] = [1, 3, 4, 6, 7, 9]).

### Input to the PCA.

A two-dimensional (2D) array was constructed for each modality (Fig. 1*B*), with each frame represented as a serialized vector containing the RGB pixel values for that frame concatenated with a serialized vector describing the local motion required to warp the current frame onto a reference frame (Fig. 1*A*; and refs. 37 and 38). This vector field was computed using a two-frame adaptation of the McGM, an optic flow algorithm modeled on the processing of the human visual system (39, 40). The arrays for each modality were then concatenated to give a multimodality hybrid array of observation by frame. Thus, each matched frame in the facial video and MR sequences was explicitly represented as a combination of the warped texture (pixel values) and the warp vector field (shape) information.

### Reconstruction and Quantification of Fidelity.

PCA was performed on the multimodal hybrid arrays. To investigate the extent to which the PCA captured shared variation between the two modalities, only one of the modalities was projected into the PCA space, and the information about both modalities was reconstructed (using all of the PCs; Fig. 1*C*). To achieve this, the pixel and warp vector values for one modality in the original multimodal array were set to zero, and then the inner product of the PCs and this partial array was computed.

To quantify the fidelity of the reconstructions, we compared reconstructed facial/MR sequences (see Fig. 1*D* for an example frame representation) with the original facial/MR sequence (Fig. 1*A*). One approach would be to correlate frame-by-frame RGB pixel values from the original and reconstructed sequences and to use the correlation coefficient as a metric for reconstruction fidelity. However, as reported previously (22), pixel correlations are not sensitive enough to provide a reliable metric of fidelity. The images across frames are inherently similar, especially in static regions such as the background, and this results in pixel correlations that are invariably high. A more appropriate metric of reconstruction fidelity is arrived at by plotting the loadings (eigenvalues) from the original PCA against those from the reconstructed representation (when only the facial video representations have been projected into the PCA space; Fig. 1*E*). A perfect reconstruction would result in all of the points lying on the unity line in Fig. 1*E*, and reconstruction fidelity can be quantified as a deviation from this pattern. The correlation between these sets of loadings, referred to here as the loading correlation, has been used previously to quantify categorization performance, as it corresponds well to similarity judgments in human observers (22).

### Selection of the Reference Frame.

To select a reference frame for each image sequence, we employed a replicable and objective procedure that involved iterating through the McGM process a number of times. On the first iteration (I_1_), the reference image was chosen randomly. The output of the McGM process consisted of vectors describing the horizontal and vertical warping necessary to project each frame to the reference and a texture vector comprised of the image on the basis of which the warp vectors would reconstruct the original image from the reference. If the McGM was perfectly accurate, this reconstructed texture vector would be identical to the reference image. The reference image for each subsequent iteration (I_*N*_) was set to the mean texture vector from the previous iteration (I_*N*__−1_). Simulations with a subset of data (for one sentence from three actors) demonstrated that both pixel and loading correlations between successive iterations converged with each iteration. Reconstruction fidelity after three iterations demonstrated low variability irrespective of the original choice of reference frame, and this variability did not diminish markedly with further iterations. Thus, for all of the data presented here, the warp vectors from the third iteration were used as input to the PCA.

### Bubbles Analysis.

To assess which regions of the video and MR sequences were important for reconstruction of the other modality, an adapted version of the Bubbles method was employed (22, 41). Each vectorized representation of the frames in the facial video sequence (the RGB pixel values and the warp vectors) was occluded using an identical mask consisting of a vectorized version of a set of randomly positioned discs (Fig. 4*A*) ensuring spatial alignment of the input data and the masks. The MR sequence was then reconstructed, as before, but using the occluded video sequence. Specifically, the 2D RGB pixel values and 2D *x* and *y* warp vectors for each video frame were separately multiplied by the 2D Boolean mask. The resulting arrays were then serialized and concatenated with the complete array for the MR frame sequence such that each frame was represented by a one-dimensional (1D) array. As before, the pixel and motion vector values for the MR sequence in the original multimodal array were set to zero, and then the inner product of the PCs and this partial array was computed. This process was repeated for 10,000 masks, with bubble positions randomly varied on each iteration. The reconstruction fidelity for each iteration was quantified as the SSE between the loadings from the original PCA (applied to the complete dataset) and the loadings from the sequence that was reconstructed using the bubble-occluded arrays. To measure the regions which led to the greatest reconstruction fidelity (*ProportionPlane)*, the bubble masks for the iterations with the lowest 10% SSE were summed together (*CorrectPlane*) and divided by the total sum of all masks (*TotalPlane*). The *ProportionPlane* was computed across the whole frame sequence and also individually for each frame in the sequence, to give a dynamic snapshot of which regions led to the highest reconstruction fidelity at each point during the sequence. We used hard-edged Boolean discs rather than the Gaussian blobs that were used originally (41) because the warp vectors were absolute and so should not be scaled. The diameter of the discs was 12 px (the full-width half-maximum of the Gaussian blobs of 5-px SD that were used previously), and the number of bubbles was twice that used previously (46 as opposed to 23 bubbles in ref. 22), to account for a doubling in the size of the images used here compared with previous work. To assess which regions of the MR sequences were important for reconstruction of the facial video sequences, *ProportionPlanes* were computed using the same process, but in this case the video sequences were reconstructed from MR sequences using the bubble-masks approach.

## Supplementary Material

Supplementary File

Supplementary File

Supplementary File

Supplementary File

Supplementary File

Supplementary File

Supplementary File

Supplementary File

Supplementary File

Supplementary File

Supplementary File

Supplementary File

Supplementary File

Supplementary File

Supplementary File

Supplementary File

Supplementary File

Supplementary File

Supplementary File

Supplementary File

Supplementary File

## Data Availability

Data and example analysis scripts can be found in Open Science Framework (OSF) at https://osf.io/3m5pr/?view_only=701b185e4b5f475-aa9057fb770e370da. Matlab data have been deposited in the OSF database (https://osf.io/) (DOI: 10.17605/OSF.IO/3M5PR).
