# Supplementary material for: The interrelationship between the face and vocal tract configuration during audiovisual speech
Source: Proc Natl Acad Sci U S A. 2020 Dec 8;117(51):32791–8. doi: 10.1073/pnas.2006192117 (PMC7768679; doi:10.1073/pnas.2006192117)
Supplement: Supplementary File [file pnas.2006192117.sapp.pdf]

PNAS
www.pnas.org

Supplementary Information for

The inter-relationship between the face and vocal tract configuration during audio-visual speech

Chris Scholes, Jeremy I. Skipper and Alan Johnston

Chris Scholes
Email: Chris.Scholes@nottingham.ac.uk

This PDF file includes:

- Legends for Movies S1 to S20
- Figures S1 and S2 with legends

Other supplementary materials for this manuscript include the following:

- Movies S1 to S20

www.pnas.org/cgi/doi/10.1073/pnas.2006192117

Movie S1-9 (separate files). Temporally aligned frontal and profile videos and MR sequence for the example sentence (1) spoken by actors 1 (S1), 4 (S2), 6 (S3), 7 (S4), 8 (S5), 10 (S6), 12 (S7), 13 (S8), and 14 (S9).

Movie S10 (separate file). Original magnetic resonance (MR) sequence (left panel), reconstructed MR sequence (middle panel) and the difference between the two (right panel), for the example sentence spoken by actor 8.

Movie S11 (separate file). The MR and video sequences reconstructed using all of the principal components (top left panels) or a single component (specified in red for each video/MR sequence pair). These sequences are for the example sentence spoken by actor 8.

Movie S12-20 (separate files). Bubble analysis *proportionPlanes* overlaid onto each frame of the video (left panel) and MR sequence (right panel) for the example sentence for actors 1 (S12), 4 (S13), 6 (S14), 7 (S15), 8 (S16), 10 (S17), 12 (S18), 13 (S19) and 14 (S20).

A note on audio – Although audio was generally recorded during both video and MR recording, facial and vocal tract sequences were aligned without using any audio, and audio played no part in the data analysis presented in the paper. Where it demonstrates good temporal alignment, audio has been added to some of the supplementary videos for illustrative purposes.

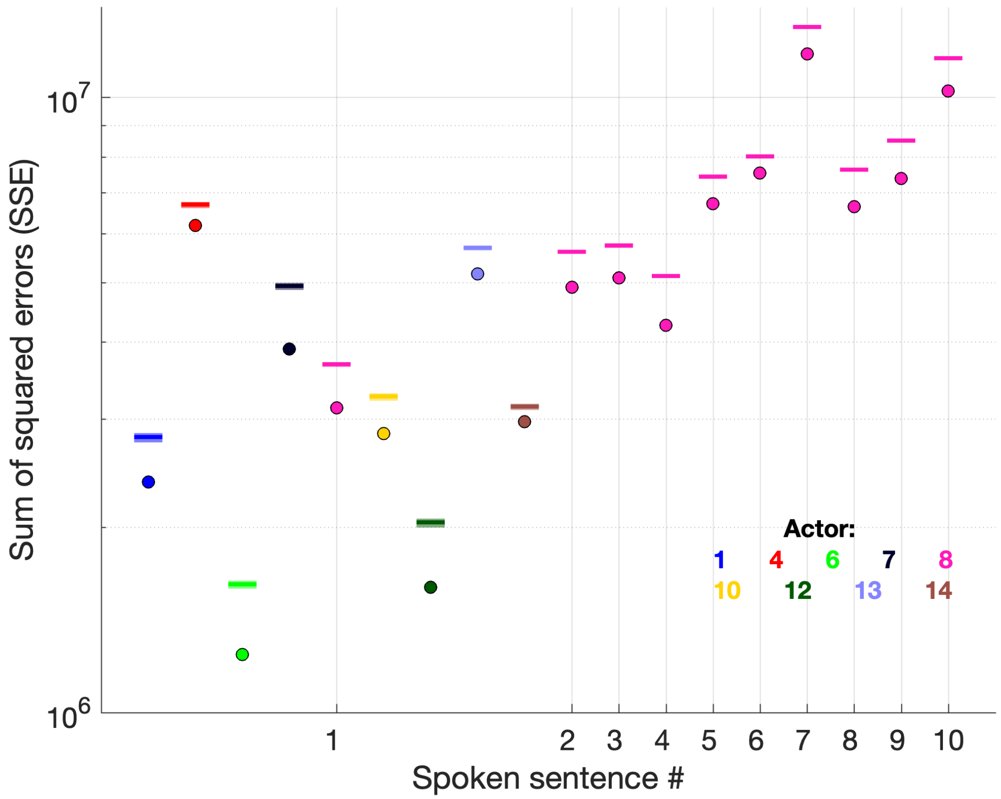

Figure S1. The sum of squared errors (SSE) between the original and reconstructed loadings for the correctly ordered sequence was always significantly lower than for the shuffled sequences. Original SSE (circles) and shuffled SSE mean (solid bar) and 95% confidence intervals (lighter bars) across all sentences for one actor and one sentence (sentence 1) for nine actors.

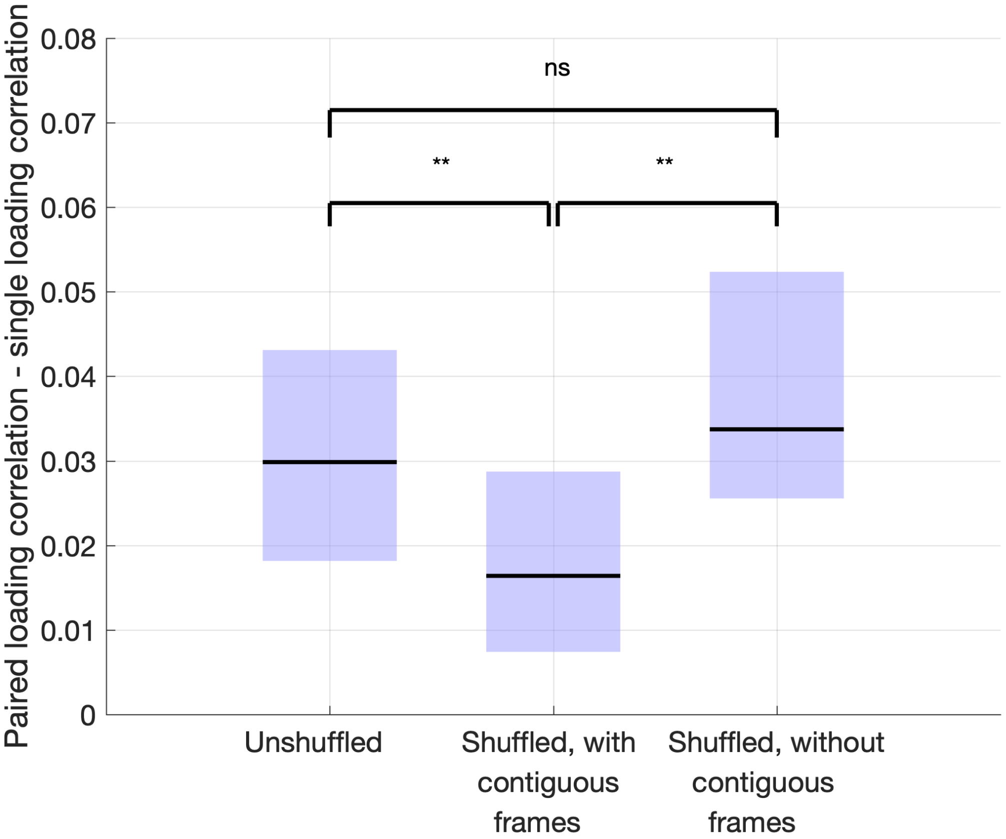

Figure S2. Implicit representation of temporal order makes paired sequences more robust to shuffling. The mean increase in loading correlation with paired versus single frames (solid lines), across actors and sentences. When contiguous frames are retained, but the order of these frame pairs is shuffled ('Shuffled, with contiguous frames' above and the squares in Figure 3b), the increase in loading correlation is lower than when the sequences are not shuffled ('Unshuffled' above and circles in Figure 3b) or when frames are shuffled *before* being paired ('Shuffled, without contiguous frames' above). A one-way ANOVA revealed significant differences between the three groups ($F(2,53) = 35.45$, $p \ll 0.01$) and a Tukey post hoc test revealed that the 'Unshuffled' and 'Shuffled, without contiguous frames' groups were not significantly different ($p = 0.18$), but that each of those groups were significantly different from the 'Shuffled, with contiguous frames' group' ($p \ll 0.01$). Shaded rectangles show 95% confidence intervals.
